# Changes in the Expression of the Toll-Like Receptor System in the Aging Rat Kidneys

**DOI:** 10.1371/journal.pone.0096351

**Published:** 2014-05-08

**Authors:** Yue Xi, Feng Shao, Xue-Yuan Bai, Guangyan Cai, Yang Lv, Xiangmei Chen

**Affiliations:** Department of Nephrology, Chinese PLA General Hospital (301 Hospital), Chinese PLA Institute of Nephrology, State Key Laboratory of Kidney Diseases (2011DAV00088), National Clinical Research Center for Kidney Diseases (2013BAI09B05), Beijing, China; Imperial College London, Chelsea & Westminster Hospital, United Kingdom

## Abstract

**Background:**

The mechanisms of kidney aging are not yet clear. Studies have shown that immunological inflammation is related to kidney aging. Toll-like receptors (TLRs) are one of the receptor types of the body's innate immune system. The function of the TLR system and the mechanisms by which it functions in renal aging remain unclear. In the present study, we, for the first time, systematically investigated the role of the TLR system and the inflammation responses activated by TLRs during kidney aging.

**Methods:**

We used western blot and immunohistochemistry to systematically analyze the changes in the expression and activation of the endogenous TLR ligands HSP70 and HMGB1, the TLRs (TLR1–TLR11), their downstream signaling pathway molecules MyD88 and Phospho-IRF-3, and the NF-κB signaling pathway molecules Phospho-IKKβ, Phospho-IκBα (NF-κB inhibition factor α), NF-κBp65, and Phospho-NF-κBp65 (activated NF-κB p65) in the kidneys of 3 months old (youth group), 12 months old (middle age group), and 24 months old (elderly group) rats. We used RT-qPCR to detect the mRNA expression changes of the proinflammatory cytokines CCL3, CCL4, CCL5, CD80, TNF-α, and IL-12b in the rat renal tissues of the various age groups.

**Results:**

We found that during kidney aging, the HSP70 and HMGB1 expression levels were significantly increased, and the expression levels of TLR1, 2, 3, 4, 5, and 11 and their downstream signaling pathway molecules MyD88 and Phospho-IRF-3 were markedly elevated. Further studies have shown that in the aging kidneys, the expression levels of the NF-κB signaling pathway molecules Phospho-IKKβ, Phospho-IκBα, NF-κBp65, and Phospho-NF-κBp65 were obviously increased, and those of the proinflammatory cytokines CCL3, CCL4, CCL5, CD80, TNF-α, and IL-12b were significantly upregulated.

**Conclusions:**

These results showed that the TLR system might play an important role during the kidney aging process maybe by activating the NF-κB signaling pathway and promoting the high expression of inflammation factors.

## Introduction

Despite the many theories of aging, such as the oxidative stress theory, that have been proposed, the mechanism of aging is not very clear[1], [2]. Recent studies have shown that immunological inflammation may be related to aging [3]. Kidneys exhibit high energy metabolism and are thus prone to aging. Kidney function in the elderly decreases with increasing age [4]. However, the mechanisms of renal aging are unclear.

Toll-like receptors (TLRs) are one of the receptor types of the innate immune system, and they play an important role in the initiation and regulation of the innate immune response and the acquired immune response. In mammals, 11 TLR family members have been identified. TLRs are a type of particular pattern recognition receptor (PRR). In addition to recognizing bacteria and other pathogenic molecules, such as lipopolysaccharides, TLRs can also recognize endogenous ligands, such as heat shock proteins (HSPs) and nucleic acid components of the body itself. After TLRs bind to their ligands, they can activate the downstream signaling adapter molecule MyD88 (myeloid differentiation factor 88) [5], further activating the NF-κB signaling pathway molecules and promoting the expression of inflammatory cytokines [6], [7]. TLR3 promotes the expression of inflammatory molecules via the activation of the MyD88-independent signaling pathway molecule IRF-3. Currently, the role of the TLR system in kidney aging is unclear.

In the present study, we, for the first time, systematically observed the changes in the expression of the TLR endogenous ligands HSP70 and HMGB1, TLR1–TLR11 and their downstream signaling pathway molecules MyD88 and IRF-3, NF-κB signaling pathway molecules, and inflammatory cytokines during the renal aging process to clarify the role of TLRs and their mechanism in kidney aging.

## Materials and Methods

### Experimental animals and specimen handling

Clean-grade inbred male F344 rats aged 2 months (weighing 180–190 g) were purchased from Beijing Vital River Laboratory Animal Technology Co., Ltd. They were divided into three groups: the 3-month-old group (youth group) (n = 20), the 12-month-old group (middle age group) (n = 20), and the 24-month-old group (elderly group) (n = 20). Three rats were kept in each cage, and they were housed in an environment at 22±1°C with 40% humidity and a 12 h photoperiod. They had free access to food and water. The rats were reared for 3, 12, or 24 months, depending on the group, and then, their body weights were measured. The rats were anesthetized with an intraperitoneal injection of sodium pentobarbital (40 mg/kg) and all efforts were made to minimize suffering, blood samples were taken, and their kidneys were removed. After rinsing the kidneys in ice-cold normal saline to remove residual blood, they were weighed. The renal tissue of the upper pole of the kidney was placed into 10% neutral formalin for fixation and used for immunohistochemistry, analysis and the remaining kidney tissue was cut into small pieces and placed into liquid nitrogen for western blot and real-time quantitative polymerase chain reaction (RT-qPCR) analysis. This study was carried out in strict accordance with the recommendations in the Guide for the Care and Use of Laboratory Animals of the Chinese PLA General Hospital. The protocol was approved by the Committee on the Ethics of Animal Experiments of the Chinese PLA General Hospital (Permit Number: X6-27).

### Biochemical analyses

The blood samples were centrifuged at 3,000 rpm for 10 min, and the sera were collected and sent to the Department of Biochemistry in our hospital to measure blood urea nitrogen (BUN), serum creatinine (SCR), glucose (GLU), triglyceride (TG) and total cholesterol (CHOL). BUN and TG were measured by colorimetric methods. Enzymatic methods were used to detect SCR and CHOL, and the hexokinase method was used to detect blood sugar. Urine samples were collected and sent to the Department of Clinical Laboratory in our hospital to detect the urinary protein/creatinine ratio, with urinary protein measured by the pyrogallol red-molybdenum method and urine creatinine measured by the sarcosine oxidase method.

### Western blot analysis

The frozen kidney tissues were lysed with a RIPA lysis buffer (50 mM Tris-Cl [pH 7.6], 150 mM NaCl, 1% NP-40, 0.1% SDS, 0.5% deoxycholic acid, 1 µg/mL leupeptin, 1 µg/mL aprotinin, and 0.5 mM phenylmethylsulfonyl fluoride) and were centrifuged at 12,000 g at 4°C for 30 min to obtain the cellular proteins in the supernatant. Equal amounts of proteins from each sample were resolved by 6%–15% SDS-PAGE, transferred to NC membranes, blocked with 5% skim milk for 1 h at room temperature, and probed with the following primary antibodies at 4°C overnight: HSP70, HMGB1,TLR1-11, MyD88, Phospho-IRF-3, Phospho-IKKβ, IκBα, NF-κBp65, Phospho-NF-κB p65 and β-actin. The detailed information on the primary antibodies used were presented in Table 1. The blots were subsequently incubated with horseradish peroxidase-conjugated anti-mouse or anti-rabbit IgG (Santa Cruz Biotechnology) at 1∶1,000–1∶5,000. Immunoreactive bands were visualized using enhanced chemiluminescence, and densitometry was performed using Quantity One software (Bio-Rad Laboratories, Hercules, California, USA).

**Table 1:** The detailed information on the primary antibodies used.

Antibody	Sources	Company	Concentration
HMGB1	Rabbit polyclonal antibody	Novus Biologicals, Littleton, CO, USA	1∶1000
HSP70	Mouse monoclonal antibody	Santa Cruz Biotechnology, Santa Cruz, CA,USA	1∶100
β-actin	Mouse monoclonal antibody	Sigma-Aldrich, St Louis, MO, USA	1∶5000
TLR1	Rabbit polyclonal antibody	Abnova, Taiwan, China	1∶500
TLR2	Rabbit monoclonal antibody	Abcam, Cambridge, UK	1∶1000
TLR3	Rabbit polyclonal antibody	Abcam, Cambridge, UK	1∶200
TLR4	Mouse monoclonal antibody	Abcam, Cambridge, UK	1∶200
TLR5	Rabbit polyclonal antibody	Abcam, Cambridge, UK	1∶500
TLR6	Rabbit polyclonal antibody	Santa Cruz Biotechnology, Santa Cruz, CA,USA	1∶250
TLR7	Rabbit polyclonal antibody	Abcam, Cambridge, UK	1∶1000
TLR9	Mouse monoclonal antibody	Abcam, Cambridge, UK	1∶200
TLR10	Rabbit polyclonal antibody	Santa Cruz Biotechnology, Santa Cruz, CA,USA	1∶200
TLR11	Rabbit polyclonal antibody	Abcam, Cambridge, UK	1∶400
MyD88	Rabbit polyclonal antibody	Santa Cruz Biotechnology, Santa Cruz, CA,USA	1∶200
Phospho-IRF-3	Rabbit polyclonal antibody	Sigma-Aldrich, St Louis, MO, USA	1∶1000
Phospho-IKKβ	Rabbit polyclonal antibody	Abcam, Cambridge, UK	1∶500
Phospho-IKBα	Mouse monoclonal antibody	Cell Signaling Techenology, Boston, USA	1∶500
NF-κBp65	Rabbit polyclonal antibody	Abcam, Cambridge, UK	1∶500
Phospho-NF-κBp65	Rabbit monoclonal antibody	Cell Signaling Techenology, Boston, USA	1∶500

### Immunohistochemistry analysis

The kidneys were fixed in 10% formaldehyde overnight at 4°C and processed for paraffin-embedding according to standard procedures. Sections were cut at 3-µm thicknesses. For immunohistochemical analyses, some tissue sections were subjected to antigen retrieval by microwaving or autoclaving for 10 or 15 min in 10 mM sodium citrate buffer (pH 6.0). Endogenous peroxidase activity was blocked by a 10-min incubation with 3% hydrogen peroxide. Sections were washed with PBS and subsequently incubated with 1.5% normal goat serum for 20 min, followed by overnight incubation with primary 1∶200 anti-TLR2 antibody at 4°C. After 3 washes with PBS, the samples were incubated with biotin-conjugated anti-IgG for 30 min at room temperature. After another wash in PBS, the sections were then incubated with a streptavidin-conjugated peroxidase (Invitrogen Corp., Carlsbad, CA, USA) for 30 min at room temperature. After a final wash in PBS, the sections were incubated with diaminobenzidine (Invitrogen) followed by microscopic examination.

### Total RNA extraction and reverse transcription

Total RNA was isolated from renal tissues using TRIzol (Invitrogen, Carlsbad,California, USA) following the manufacturer's instructions. A UV spectrophotometer was used to measure the concentrations of total RNA. Reverse transcription was performed using a TIANScript RT kit (Tiangen Biotech, Beijing, China).

### Real time-quantitative polymerase chain reaction (RT-qPCR)

Amplification was performed in a 7500 real-time PCR System (Applied Biosystems, Foster, California, USA). The reaction contained 50 ng of cDNA, 0.2 µM primers, and 10 µL of 2 Χ SYBR green buffer (TaKaRa, Dalian, Liaoning, China) in a final volume of 20 µL. The primers (Table 2) were designed using the software package Primer Express 2.0 (Applied Biosystems, Foster, California, USA) based on GenBank nucleotide sequences. PCR was performed using the following cycling conditions: 95°C for 30 s and 40 cycles of denaturation at 95°C for 15 s and extension at 60°C for 30 s. All samples were run in triplicate. The relative abundance of target mRNA was determined with the comparative cycle threshold method [8].

**Table 2:** RT-qPCR primer sequences and the lengths of the products.

Name	Genbank No.	Primers	Length of products
IL-6	NM_012589	F: ATTGTATGAACAGCGATGATGCAC R: CCAGGTAGAAACGGAACTCCAGA	150 bp
CCL3	U06435	F:ATATGGAGCTGACACCCCGA R:GTCAGGAAAATGACACCCGGC	123 bp
CCL4	U06434	F: CTTCTGCGATTCAGTGCTGTC R:AGCAAAGGCTGCTGGTCTCA	128 bp
CCL5	NM_031116	F: ACCAGCAGCAAGTGCTCCAA R:AGCTGGTTAGGACTAGAGCAAGCAA	189 bp
ICAM-1	NM_012967	F:TGAGCGACATTGGGGAAGAC R:TCGCTCTGGGAACGAATACAC	104 bp
CD80	NM_012926	F: TCGTACGTGGTGAAACACCTGA R:CCGGAAGCAAAGCAGGTAATC	125 bp
TNF-α	NM_012675	F: ATACACTGGCCCGAGGCAAC R: CCACATCTCGGATCATGCTTTC	75 bp
IL-12b	NM_022611	F:TGGGAGTACCCTGACTCCTG R:GAGGAACGCACCTTTCTGGT	129 bp
GAPDH	NM_017008	F: GAGAAGGCTGGGGCTCAC R:GTTGTCATGGATGACCTTGGC	186 bp

F, forward; R, reverse; IL-6: interleukin-6; CCL3: chemokine ligand 3; CCL4: chemokine ligand 4; CCL5: chemokine ligand 5; ICAM-1: intercellular adhesion molecule-1; TNF-α: tumor necrosis factor-α; IL-12b: interleukin-12b; GAPDH: glyceraldehyde-3-phosphate dehydrogenase (internal reference).

### Statistical methods

All data were analyzed using the SPSS 17.0 (SPSS, Chicago, Illinois, USA) software package. Comparisons between the data of various groups were performed using analyses of variance with P values <0.05 indicating significant differences.

## Results

### Changes in renal function in the aging rats

First, we examined the changes in the renal and metabolic function parameters in the rats in the 3-month-old, 12-month-old, and 24-month-old groups. We found that compared with the 3-month-old group, the 12-month-old and 24-month-old groups showed significant increases in body weight, kidney weight, triglyceride, and the urinary protein/creatinine ratio; blood urea nitrogen levels were significantly increased in rats of the 24-month-old group, while serum creatinine, glucose, and total cholesterol levels did not change significantly. Compared with the 12-month-old group, the 24-month-old group showed significantly increased kidney weight and urinary protein/creatinine ratios (Table 3).

**Table 3:** Changes in the kidney function and metabolic function in the aging rats.

Parameters	3 month	12 month	24 month
Body Weight (g)	236.3±12.28	466.86±32.62*	617.8±57.34*
Kidney weight (g)	1.67±0.68	3.250±0.5*	5.01±1.0* #
Blood urea nitrogen (mmol/L)	5.73±0.47	6.51±0.40	6.86±1.10*
Serum creatinine (µmol/L)	27.15±2.60	25.62±1.94	25.8±2.04
Serum glucose (mmol/L)	7.09±1.02	5.745±0.29	6.09±1.31
Serum triglyceride (mmol/L)	1.20±0.32	2.32±0.46*	3.22±0.82*
Serum cholesterol (mmol/L)	1.55±0.16	2.73±0.28	3.11±0.36
Urine protein/creatinineratio (mg/mmol)	146.01±22.72	164.81±38.31*	256±49.39* #

Compared with the 3-month-old group;

* indicates P<0.05; compared with the 12-month-old group;

# indicates P<0.05.

### Changes in the expression of endogenous ligands of TLRs in the aging rat kidney tissues

**Figure 1:**
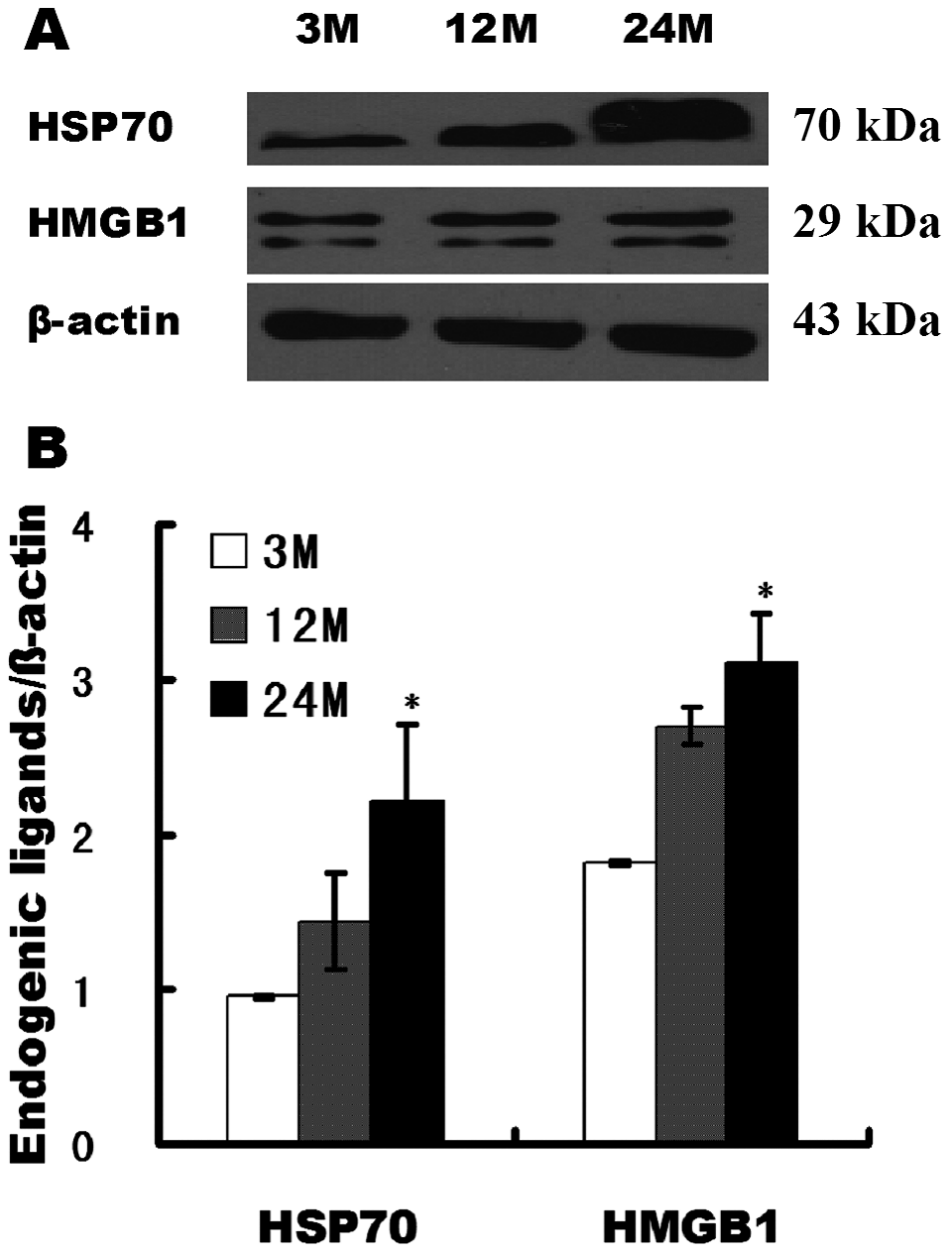
Expression of endogenous ligands of TLRs in rat kidney tissues in different age groups. (**A**) Western blot detection of the HSP70 and HMGB1 protein expression levels in various groups of rats. (**B**) Quantitative analysis of gray scales for the HMGB1 and HSP70 expression levels. β-actin: internal reference. Compared with the 3-month-old group, * indicates P<0.05.

HSP70 and HMGB1 are endogenous ligands of TLRs and can bind to TLRs and activate downstream signaling pathways and inflammatory responses. We used western blotting to detect the changes in the expression of the HSP70 and HMGB1 proteins in the kidney tissues of rats in different age groups. Compared with the 3-month-old group, expression levels of the HMGB1 and HSP70 protein were significantly increased in the 12-month-old and 24-month-old groups (Figure 1).

### Expression changes of the MyD88-dependent TLRs in the kidney tissue of aging rats

**Figure 2:**
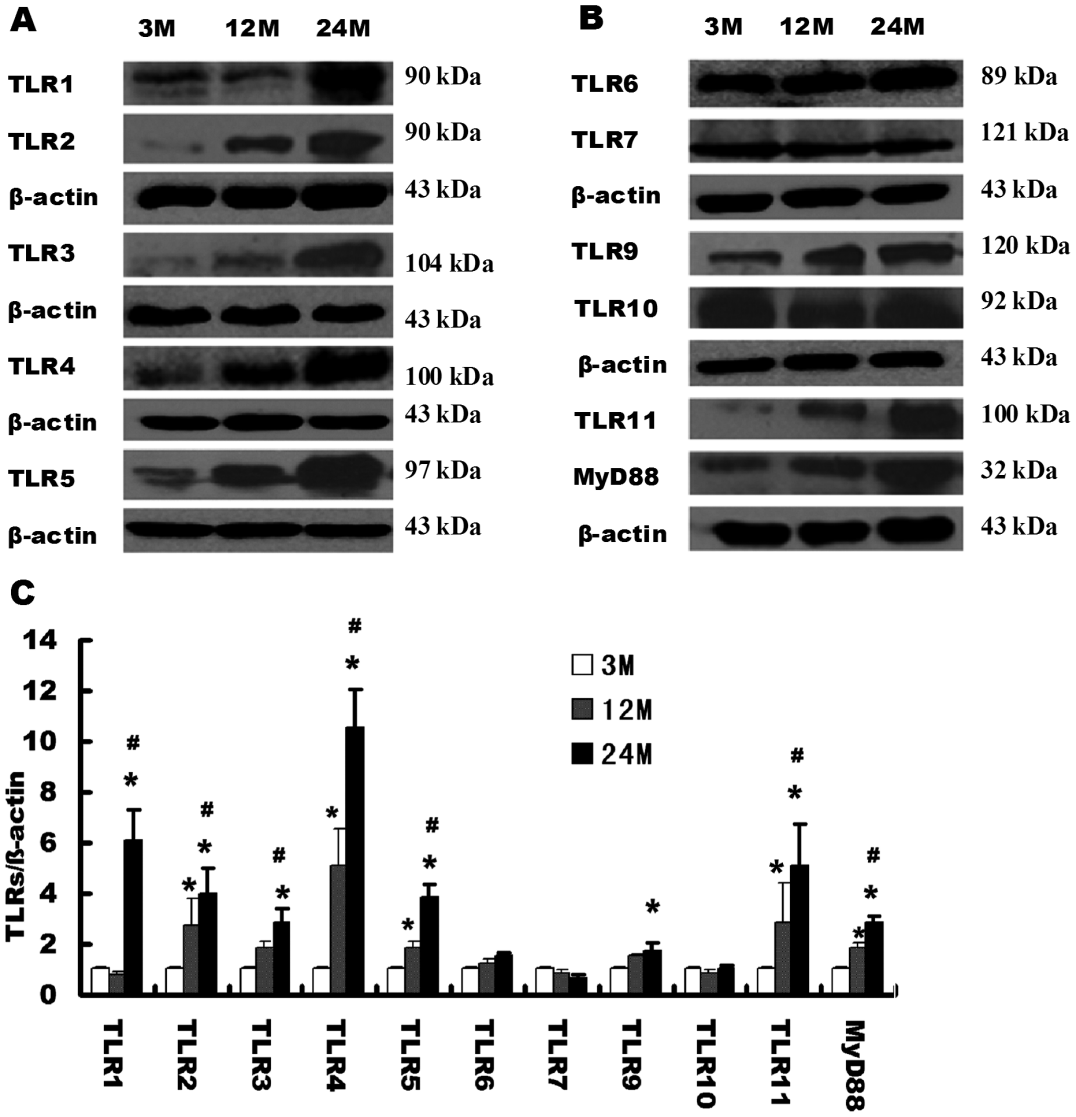
Expression analysis of MyD88-dependent TLRs in the renal tissues of rats in different age groups. (**A**) Western blotting was used to detect the expression levels of TLRs in each group of rats. (**B**) Quantitative gray scale analysis of TLR levels. β-actin: internal reference. Compared with the 3-month-old group, * indicates P<0.05; compared with the 12-month-old group, # indicates P<0.05.

Except for TLR3, all TLRs can activate the MyD88-dependent signaling pathway after binding with ligands. TLR4 can activate both the MyD88-dependent signaling pathway and the MyD88-independent pathway. We used western blotting to detect expression changes of TLR family members in the rat renal tissues of all age groups. Compared with the 3-month-old group, the expression levels of TLR2, 4, 5, and 11 were significantly elevated in the renal tissues of the 12-month-old and 24-month-old groups. TLR1 was only significantly increased in the 24-month-old group, and TLR9 slightly increased in the 24-month-old group. TLR6, 7, and 10 showed no significant changes, and TLR8 was not detected. Compared with the 12-month-old group, TLR1, 2, 3, 4, 5, and 11 were significantly increased in the 24-month-old group (Figure 2). We found that the expression level of the TLR downstream signaling pathway adapter molecule MyD88 increased significantly with increasing age.

**Figure 3:**
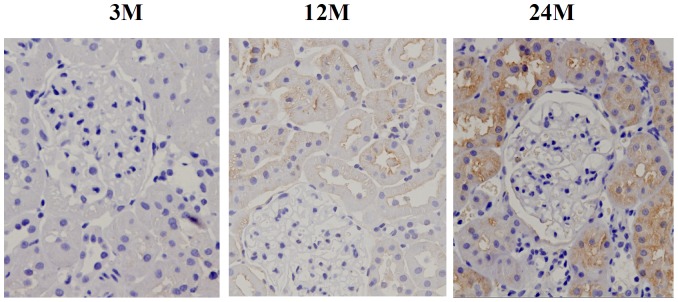
Immunohistochemical analysis of TLR2 expression level in rat renal tissues in different age groups.

We further analyzed the cellular location and expression level of TLR2, a representive of TLRs using immunohistochemistry technique. The results demonstrated that TLR2 was mainly expressed on the cellular membrane of renal tubular epithelial cells (Figure 3). The expression level of TLR2 was elevated in the renal tissues of the 12-month-old and 24-month-old groups, compared with the 3-month-old group (Figure 3).

### Expression and activation of MyD88-independent TLRs and its downstream pathway molecule IRF-3 in the aging kidneys

**Figure 4:**
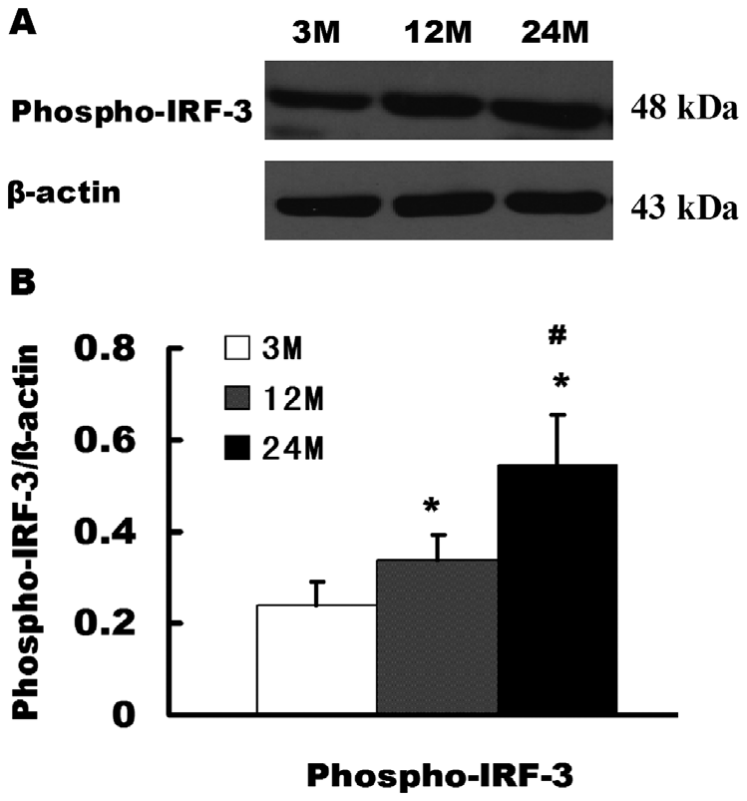
Activation changes of the MyD88-independent downstream signaling pathway molecule IRF-3 in the aging kidneys. (**A**) Western blotting was used to detect the expression levels of the Phospho-IRF-3 protein in various groups of rats. (**B**) Quantitative gray scale analysis results. Compared with the 3-month-old group, * indicates P<0.05; compared with the 12-month-old group, # indicates P<0.05.

Western blot was used to detect the expression and activation of TLR3 and its downstream signaling pathway molecule IRF-3 in the kidneys of rats in different age groups. Compared with the 3-month-old group, the 12-month-old and 24-month-old groups showed significantly increased TLR3 expression levels. Compared with the 12-month-old group, TLR3 was significantly higher in the 24-month-old group (Figure 2). Compared with the 3-month-old group, Phospho-IRF-3 (activated IRF-3) levels were significantly increased in the 12-month-old and 24-month-old groups, and compared with the 12-month-old group, Phospho-IRF-3 levels were significantly higher in the 24-month-old group (Figure 4).

### Expression changes in members of the NF-κB signaling pathway in the aging rat kidney tissues

**Figure 5:**
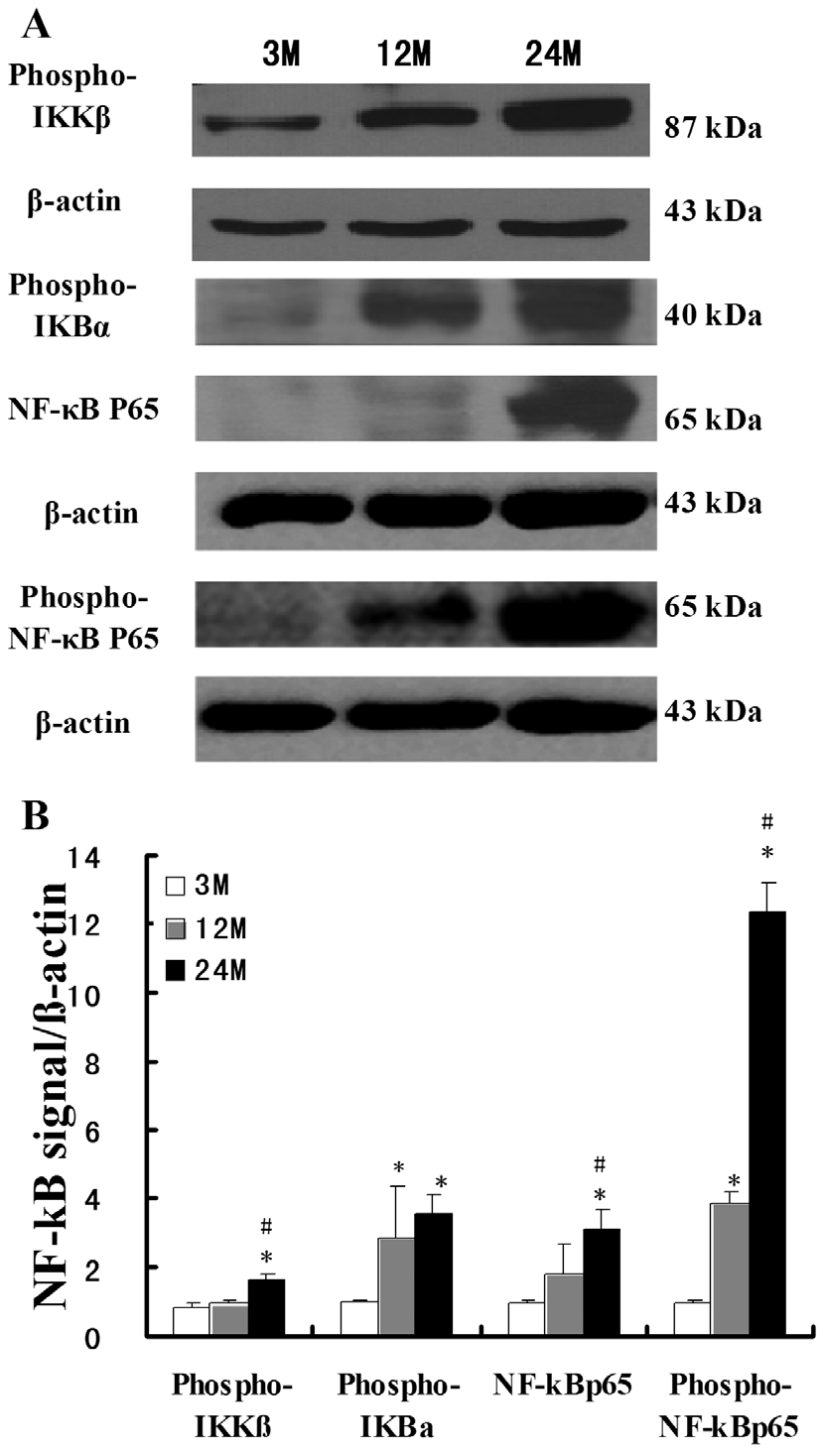
Expression of NF-κB signaling pathway molecules in rat kidney tissues in different age groups. (**A**) Western blot detection of the expression of NF-κB signaling pathway proteins in various rat groups. (**B**) Quantitative analysis of gray scale. Compared with the 3-month-old group, * indicates P<0.05; compared with the 12-month-old group, # indicates P<0.05.

NF-κB is a key transcription factor that regulates the intracellular expression of various proinflammatory cytokines. Western blot analysis was used to detect the expression changes of the NF-κB signaling pathway molecules. Compared with the 3-month-old group, Phospho-IKKβ (activated IKKβ) was significantly increased in the 24-month-old group. Compared with the 12-month-old group, the 24-month-old group showed significantly increased Phospho-IKKβ. Compared with the 3-month-old group, Phospho-IKBα and Phospho-NF-κBp65 (activated NF-κBp65) showed significant increases in the 12-month-old and 24-month-old groups, and NF-κBp65 showed significant increases only in the 24-month-old group. Compared with the 12-month-old group, NF-κBp65 and Phospho-NF-κBp65 showed significant increases in the 24-month-old group (Figure 5).

### Expression changes in inflammatory factors in the aging rat kidney tissues

**Figure 6:**
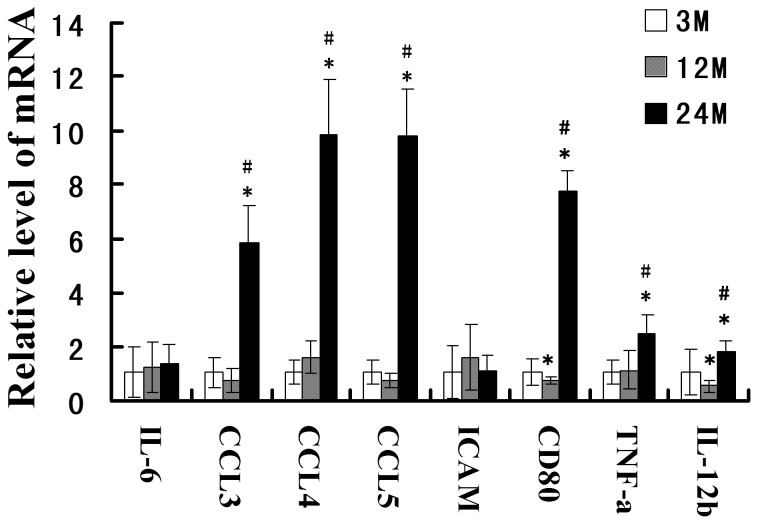
RT-qPCR detection of the expression of inflammatory cytokines in rat renal tissues in different age groups. Compared with the 3-month-old group, the fold change in inflammatory cytokines in the 12-month-old and 24-month-old groups  = 2^−ΔΔCT^. Compared with the 3-month-old group, * indicates P<0.05; compared with the 12-month-old group, # indicates P<0.05.

We then used RT-qPCR to detect mRNA expression changes of the inflammatory cytokines IL-6, CCL3, CCL4, CCL5, ICAM-1, CD80, TNF-α, and IL-12b in the renal tissues of rats in different age groups. Compared with the 3-month-old group, the expression levels of CCL3, CCL4, CCL5, CD80, TNF-α, and IL-12b were significantly increased in the 24-month-old group. The CD80 and IL-12b expression levels were slightly reduced in the 12-month-old group, but the remaining differences were not significant. Compared with the 12-month-old group, the CCL3, CCL4, CCL5, CD80, TNF-α, and IL-12b expression levels were also significantly increased in the 24-month-old group (Figure 6).

## Discussion

At present, the role of the inflammatory molecules during the process of organ aging and aging-related diseases is not clear. There two theories have been proposed, i.e. oxidative stress theory and cytokine theory. Accumulated data suggest that during the aging process of organism, excessive oxidative stress may activate many pro-inflammatory signaling pathways, including the NF-kB signaling pathway, and induce continuous (chronic) up-regulation of pro-inflammatory mediators (e.g., TNF-a, IL-1b, IL-6, COX-2, iNOS, etc.), leading to “inflamm-aging” of tissues and organs [9]–[11]. In recent years, studies have proved that senescence at a cellular level is directly linked to an interleukin (IL)-dependent inflammatory network. IL-6 and IL-8, two well-known proinflammatory cytokines, seem to play a central role in induction of premature cellular senescence. Activation of the above-mentioned molecules and their receptors is necessary for the initiation of senescence while their deactivation ceases the process [12]. Thus, in the future, the organ aging may be delayed by blocking activation of inflammatory signaling pathway.

Structural changes in the aging kidneys include focal segmental glomerular sclerosis (accumulation of extracellular matrix such as collagen), atrophy of renal tubules, interstitial fibrosis (accumulation of extracellular matrix such as collagen), infiltration of inflammatory cells (macrophage, etc.), activation of myofibroblasts, and an increased expression of inflammatory molecules including pro-inflammatory cytokine (TNF-α, ILs, etc.), chemokines, adhesion molecules, etc. Functional alterations in the aging kidneys include decreased GFR, decreased renal plasma flow, decreased functional reserve, impaired urine dilution/concentration ability, impaired Na+K+handling, and impaired oxidants handling [13].

The underlying mechanisms that inflammatory molecules impact the age-associated renal structure and functional remodeling are not clear at present. Study on an animal model found that an inflammatory cytokine, TGF-beta, may activate fibroblasts into myofibroblasts, which may synthetize a lot of collagens and lead to glomerular sclerosis and interstitial fibrosis. TGF-beta also induces prominent proliferation of renal cells such as glomerular mesangial cells and interstitial fibroblasts by Abelson nonreceptor tyrosine-kinase (c-abl) and p21–activated kinase-2 (PAK2) pathway. Increased TNF-α expression may induce apoptosis of renal tubular cells [14], [15].

The innate immune system was first discovered due to its ability to initiate inflammation and immune responses, eliminate infections, and repair damaged tissues. TLRs play an important role in the induction of innate and adaptive immunity. TLRs are widely expressed in immune cells, such as dendritic cells, neutrophils, monocytes, T cells, and B cells, and they can also be expressed in cells of many tissues and organs. Renal tubular epithelial cells can express TLR1–TLR4, and TLR6 mRNAs. TLRs can also be expressed in renal glomerular cells. For example, TLR4 mRNA can be expressed in podocytes, Bowman's capsule cells, and mesangial cells [16].

After TLRs (except TLR3) bind with pathogenic molecules, they can activate the downstream signaling molecule MyD88 by forming dimers [17]. In the resting state, IκBα binds with NF-κB and inhibits it so that it stays in a resting state in the cytoplasm. After activation, MyD88 can further activate IKKβ, which leads to the ubiquitination of IκBα and the release of IκBα from IκB/NF-κB complexes. Subsequently, NF-κB is activated and translocated into the nucleus, thereby activating the expression of a series of proinflammatory cytokine genes, such as IL-6, CCL3, CCL4, CCL5, ICAM-1, CD80, TNF-α, and IL-12b [18], [19]. TLR3 can activate the MyD88-independent signaling pathway molecule IRF-3 and then activate the expression of inflammatory cytokines such as IFN-β.

Recent studies have found that TLRs are closely associated with kidney diseases [20]. TLR7 and TLR9 expression levels are significantly elevated in glomerulonephritis in systemic lupus erythematosus [21]. TLR2 and TLR4 play an important role in ischemia-reperfusion renal injury. Studies have found that TLR2-deficient mice can avoid functional and structural ischemic injuries [22]. TLR1 and TLR9 expression levels are increased in apoferritin-induced nephritis, and TLR9 polymorphisms are related to many human diseases, including IgA nephropathy [23]. Ligands of TLR3 may induce the expression of CD80 in human renal podocytes by binding with TLR3 and activating downstream signaling pathways [24]. TLR2 and TLR4 play an important role in diabetic nephropathy, and especially in the high-fat diet-induced diabetes rat model, TLR4 and its downstream IKKβ/NF-κB pathway components are significantly activated [25]–[27]. However, currently, the roles of TLRs in the renal aging process remain unclear.

In the present study, we, for the first time, systematically investigated the expression levels of the endogenous ligands of TLRs and TLR signaling pathway molecules in the process of rat kidney aging. In the aging kidney, the expression levels of the endogenous TLR ligands HSP70 and HMGB1 were significantly increased, as were those of TLR1, 2, 3, 4, 5, and 11. We then examined the expression and activation of the TLR signaling pathway downstream molecules MyD88 and IRF-3 and found that MyD88 and Phospho-IRF-3 levels were significantly elevated in the aging kidneys.

NF-κB is a key transcription factor that regulates the intracellular expression of various proinflammatory cytokines. After activation, TLRs can activate the NF-κB signaling pathway. In the present study, we found that in the aging kidneys, the expression levels of the NF-κB signaling pathway molecules Phospho-IKKβ, Phospho-IκBα, NF-κBp65, and Phospho-NF-κBp65 were significantly increased. Accordingly, we also found that the expression levels of the proinflammatory cytokines CCL3, CCL4, CCL5, CD80, TNF-α, and IL-12b were significantly upregulated in the aging kidney tissues. These results show that the TLR system may activate the NF-κB signaling pathway and promote the expression of inflammation factors, thereby playing an important role in the aging process of the kidneys.

Recent studies have shown that immunological inflammation may be associated with aging and aging-related diseases. Serum levels of inflammatory cytokines, such as TNF-α, IL-6, and C-reactive protein (CRP), are significantly increased in elderly populations. However, the mechanisms by which inflammation is involved in tissue and organ aging are not very clear. The present study showed that the TLR system was closely related to the aging of the kidneys, which provides a new direction for the further investigation of the renal aging mechanism.
